# Analysis of Medication Errors Reported by Community Pharmacists in the Republic of Korea: A Cross-Sectional Study

**DOI:** 10.3390/medicina59010151

**Published:** 2023-01-12

**Authors:** Ju-Hee Han, Kyu-Nam Heo, JiMin Han, Mo-Se Lee, Su-Jin Kim, Sangil Min, Young-Mi Ah, Ju-Yeun Lee

**Affiliations:** 1 College of Pharmacy, Seoul National University, Seoul 08826, Republic of Korea; 2 College of Pharmacy, Chungbuk National University, Cheongju 28160, Republic of Korea; 3 Regional Patient Safety Center, Korean Pharmaceutical Association, Seoul 03080, Republic of Korea; 4 Department of Surgery, Seoul National University Hospital, Seoul 03080, Republic of Korea; 5 Quality and Patient Safety Center, Seoul National University Hospital, Seoul 03080, Republic of Korea; 6 College of Pharmacy, Yeungnam University, Gyeongsan-si 38541, Republic of Korea

**Keywords:** medication error, community pharmacy, high-risk medications, patient safety

## Abstract

*Background and objectives*: We aimed to describe medication-related incidents or medication errors (MEs) reported by community pharmacists and analyze the prevalent medications involved. *Materials and Methods*: We extracted ME reports from databases comprising patient safety incidents reported to the Korean Pharmaceutical Association between January 2013 and June 2021. Medications were analyzed according to the second (therapeutic subgroup) and fifth (chemical substance) levels of the Anatomical Therapeutic Chemical classification. *Results*: A total of 9046 MEs were identified, most of which were near miss reports (88.3%). Among the errors that reached the patients (521 cases), harmful incidents accounted for 76.8%. Most MEs occurred during prescription (89.5%), while harmful MEs occurred mainly during dispensing (73.3%). In the prescription step, wrong drugs (44.8%), dosing errors (27.0%), and wrong durations (14.0%) were common. Anti-inflammatory and anti-rheumatic products (M01), drugs for acid-related disorders (A02), and antihistamines for systemic use (R06) were the most frequently reported medication classes involved. Harmful incidents were most common for dosing errors (31.0%) and wrong drugs (26.8%) and were common with warfarin, levothyroxine, and glimepiride. *Conclusions*: The MEs reported by community pharmacists were mainly prescribing errors, most of which were rectified before reaching patients. The prevalent medications involved in harmful errors include anti-diabetic, anti-thrombotic, and anti-inflammatory agents.

## 1. Introduction

Medication errors (MEs) are recognized worldwide as a major problem in healthcare systems [1] because they can increase morbidity, mortality, and costs. In the United States alone, MEs have been estimated to cause at least one death per day and injure 1.3 million people every year [2]. In addition, USD 42 billion in ME-related costs is incurred each year [2]. Against this background, the World Health Organization (WHO) selected “Medication Without Harm” as the theme of the third Global Patient Safety Challenge in 2017 and aimed to reduce medication-related harm by 50% over the next five years [2].

The type of ME can be classified according to how it occurred as follows: wrong patient, wrong drug, wrong dose, wrong route, omission, etc. Additionally, MEs can be classified based on contextual information such as time, place, medicine, and people [3,4]. Differences in the type of clinical problem, classes of drugs used, and service composition may lead to the difference in the main types of ME observed between inpatient and outpatient settings [5]. A study that analyzed ME patterns in elderly patients in France showed that antibacterials for systemic use, anti-neoplastic agents, and wrong techniques were most frequently involved in MEs occurring within a hospital setting, while psycholeptics, anti-diabetic drugs, and prescribing to wrong patients were most frequently involved in MEs within a community setting [6]. 

Reporting and learning systems play important roles in reducing the overall occurrence of MEs. Analyzing ME incident reports makes it possible to identify risk factors, prioritize problems, and establish effective prevention strategies [7]. However, the analysis of MEs has been performed in only limited studies in Korea to date. Most studies have been related to attitude to or perception about the reporting of MEs [8,9] and were limited to cases of adverse drug events or were reported in the hospital setting [10,11]. Woo et al. reported characteristics of MEs using the database of the Korea Adverse Events Reporting System. However, they focused on pediatric patients, and only 208 MEs were included [12]. Analyses of MEs in Korea with a focus on the community setting have not been reported.

Since the Patient Safety Act came into force in the Republic of Korea in 2017, patient safety incidents have been reported to a governmental agency: the Korea Institute for Healthcare Accreditation [13]. Accordingly, the Korean Pharmaceutical Association (KPA) has established a system called the “KPA Safe Pharm System” to collect reports of adverse drug events and patient safety incidents from community pharmacists nationwide [14]. This system is characterized by its connection with a routinely used billing program. To encourage and facilitate reporting by pharmacists, the system allows them to report incidents conveniently and quickly during their daily work [14].

Here, we have aimed to describe the medication-related incidents or MEs reported by community pharmacists and analyze the prevalent medications involved using data gathered through the KPA Safe Pharm System covering community pharmacies nationwide.

## 2. Methods

**Figure 1:**
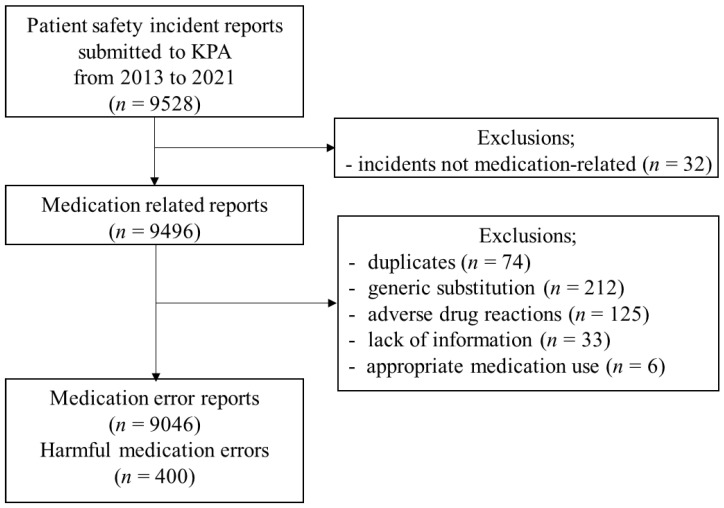
Medication error case selection process. KPA, Korean Pharmaceutical Association.

In this cross-sectional study, we combined and analyzed two databases: (1) an anonymized database of pharmacists’ liability claims received by the KPA through a community pharmacy group professional liability insurance program between 2013 and February 2021, and (2) a database that gathered patient safety incidents and MEs through the KPA Safe Pharm System between 2018 and June 2021. The pharmacist liability claims database included free text descriptions containing the location, date, incident, and related medications. The Safe Pharm reports were structured with patient information, incidents of erroneous situations, and related medications. Non-medication-related incidents, duplicated reports, intended generic substitutions, and adverse drug reactions to appropriate drug use processes were excluded (Figure 1). We performed this study following the Strengthening the Reporting of Observational Studies in Epidemiology statement for cross-sectional studies [15].

We used the definition of ME employed by the National Coordinating Council for Medication Error Reporting and Prevention (NCC MERP): “any preventable event that may cause or lead to inappropriate medication use or patient harm while the medication is in the control of the health care professional, patient, or consumer” [16]. We included both near misses blocked before reaching the patients and MEs that caused harm to patients.

Our principal aim was to identify the key steps, error types, and medications involved in MEs in community pharmacies. To this end, the researchers reviewed all medication error incident reports and extracted the following information: medication use process, type of error, level of patient harm, and medications involved [17]. Instances where any of these aspects could not be identified in the reports were classified as “missing”. We separately analyzed the characteristics of the harmful medication errors that reached the patient (non-near miss) and caused harm.

The medication use process was classified into prescribing, dispensing (including the steps guiding on how to take and give medications to patients), and administration (the step in which patients take the medications) according to the suggestions of the Institute of Medicine (IOM) report [18]. The type of error was classified using a modified version that can be applied to the community in consideration of the WHO Conceptual Framework for the International Classification for Patient Safety (ICPS) [4] and the NCC MERP taxonomy of medication errors (Supplementary Table S1) [19].

Referring to the revised form of the Patient Safety Act in Korea, the level of harm to the patient was classified as near miss, no harm, mild harm (temporary injury requiring short-term intervention), moderate harm (long-term injury requiring extended hospitalization or additional treatment), severe harm (permanent injury requiring intervention or acute life-saving treatment), and death.

Medications involved in MEs were coded as Anatomical Therapy Chemical (ATC) Classification System levels 2 and 5. Cases in which a specific medication could not be identified were excluded when analyzing medications related to MEs. Descriptive statistics are given as frequencies and percentages.

## 3. Results

We identified 9046 MEs from patient incident reports submitted to the KPA between 1 January 2013 and 30 June 2021 (Figure 1). The majority of MEs were near misses (88.3%), intercepted by community pharmacists or patients before the medication could be taken. Of the 521 MEs that reached the patient, 400 (76.8%) were harmful. MEs that caused moderate to severe harm accounted for 1.6% of all cases (Table 1).

In terms of medication use, the prescribing stage (89.5%) accounted for the majority of overall reported MEs, followed by the dispensing (6.7%) and administering stages (1.9%) (Table 1). On the other hand, the most common process involved in harmful MEs was dispensing (73.3%), followed by administering (11.0%) and prescribing (10.0%) (Table 2).

MEs were categorized into ten types of error. In MEs of the prescribing phase, “wrong drug” (*n* = 3628, 44.8%) was the most frequently reported, followed by “dosing error” (*n* = 2193, 27.1%) and “wrong duration” (*n* = 1136, 14.0%). In the dispensing and administering phases, “dosing error” was the most frequently reported (*n* = 153, 25.3% and *n* = 86, 50.3%, respectively), followed by “wrong drug” (*n* = 149, 24.6% and *n* = 27, 15.8%, respectively). The “wrong drug” error mainly involved therapeutic duplication (*n* = 2629, 68.9%), followed by different drugs (*n* = 613, 16.1%) and known drug allergies (*n* = 148, 3.9%) (Table 1).

Regarding the overall harmful error, “dosing error” (*n* = 124, 31.0%) was the most frequently reported, followed by “wrong drug” (*n* = 107, 26.8%) and “wrong patient” (*n* = 22, 5.5%). However, in each of the prescribing and dispensing phases, “wrong drug” (*n* = 20, 50.0% and *n* = 86, 29.4%, respectively) was the most frequently reported, followed by “dosing error” (*n* = 9, 22.5% and *n* = 83, 28.3%, respectively). In the administration phase, more than half of the reports were on “dosing error” (*n* = 27, 61.4%). The MEs causing severe harm mainly involved “dosing error” in the dispensing stage. (Table 2)

**Figure 2:**
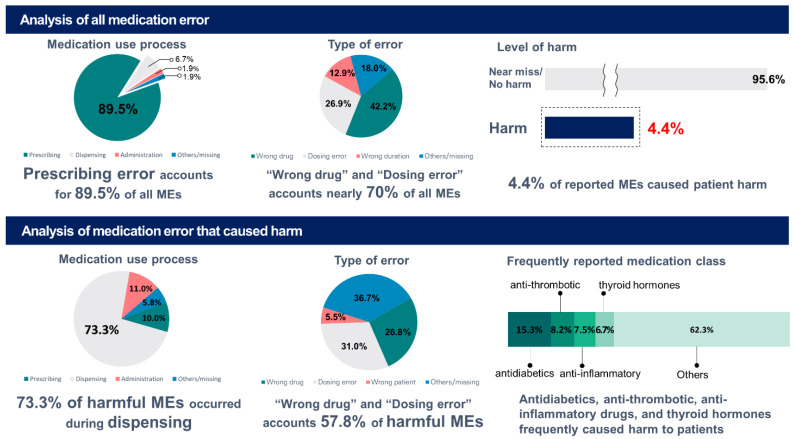
Diagrammatic summary of study findings.

A total of 599 and 137 chemical substances in 75 and 46 therapeutic subgroups, respectively, were involved in the overall and harmful MEs. Anti-inflammatory and anti-rheumatic products (M01, *n* = 1518, 18.8%), drugs for acid-related disorders (A02, *n* = 813, 10.1%), and antihistamines for systemic use (R06, *n* = 770, 9.5%) were the most commonly reported therapeutic categories of MEs. The most reported medication was loxoprofen (*n* = 940, 11.6%), followed by bepotastine (*n* = 331, 4.1%) and mosapride (*n* = 193, 2.4%) (Supplementary Table S2). In terms of harmful MEs, the most frequently reported medication classes were anti-diabetic drugs (A10), anti-thrombotic agents (B01), and anti-inflammatory and anti-rheumatic products (M01) (Table 3). In addition, warfarin, levothyroxine, and glimepiride were the most frequently reported drugs that caused harm. Severe cases of harmful MEs were caused by anti-hypertensive drugs (*n* = 2), anti-Parkinson drugs (levodopa, carbidopa, and entacapone; *n* = 1), and anti-thrombotic agents (dabigatran; *n* = 1). A summary of the findings observed is presented in Figure 2.

## 4. Discussion

In this nationwide cross-sectional study, we analyzed MEs reported by community pharmacists. We found that most of the reported MEs occurred during the prescription process, consistent with the reports of previous studies [20,21,22]. These findings might have been affected by the reporters, mainly pharmacists, and outpatient settings. Previous studies have shown that administration was the most commonly reported process in the hospital setting, followed by prescription and dispensing [23,24]. This difference may be explained by the frequent use of injectable medications that are susceptible to administration errors, differences in the capacity of different settings to better detect MEs and harm through direct monitoring, and differences in the established systems for reporting MEs in hospitals compared with community settings [25]. Nevertheless, these results suggest that MEs are common during the prescribing stage and that strategies to enhance effective prescription reviews and interventions by community pharmacists are needed.

Although the dispensing error rate in community pharmacies is known to be low at 0.015 [26], considering that more than 400 million prescriptions are dispensed by community pharmacists in Korea every year [14], we could not exclude the possibility of underreporting of dispensing errors in this study. Although the KPA has been conducting educational activities to promote ME reporting by community pharmacists since 2018 [14], it is necessary to continue to strive to obtain high-quality reports, including more complete reporting of dispensing errors.

In line with previous reports, the most prevalent types of MEs in our study were “dosing error” and “wrong drug” [6,21,27]. Previous studies that analyzed dispensing errors of community pharmacists showed that incorrect doses, drugs, and quantities were the most frequently reported types of errors [28].

Similarly to previous studies that analyzed ME reporting data [21,29], most reported MEs were near misses. This finding suggests that community pharmacists play an important role in preventing errors from reaching patients by intervening at the level of prescription errors with close to 90% effectiveness [30]. While many prescription errors were prevented by pharmacists from harming the patients, the majority of pharmacists’ dispensing errors did reach the patients, and some caused harmful events. In such situations, the patient’s active participation in treatment and providing thorough patient counseling can go a long way towards preventing MEs, along with many strategies to reduce dispensing errors in pharmacies. Patients who are educated about their condition and counselled about their medication are often in a better position to identify and block prescription or dispensing errors [29,30].

In our study, anti-inflammatory analgesics, drugs for acid disorders, antihistamines, cold medications, and antibacterials were the most frequently reported classes of medication. Similarly, another study reported that the most common drug classes in out-of-hospital MEs included cough/cold medications, analgesics, cardiovascular agents, antihistamines, antidepressants, and antimicrobial agents [31]. We also found that anti-thrombotic agents including warfarin, thyroid hormone, and anti-diabetic agents including glimepiride were medications frequently involved in harmful MEs. These medications are vulnerable to medication errors because they are marketed in various tablet strengths, and they bear an increased risk of causing serious patient harm when they are used incorrectly. Reflecting these facts, they are included in the ISMP List of High-Alert Medications in Community/Ambulatory Healthcare [32]. Therefore, developing and implementing best-practice guidelines on managing these medications during all medication processes, especially in the dispensing stage, can be an effective strategy to reduce drug-related harm in the community.

Considering that most harmful medication errors occurred in the dispensing stage, and the main types of MEs were dosing errors and wrong drug errors, efforts for systemic improvements in community pharmacies are needed to prevent dispensing errors for these medications. Individual community pharmacies could consider the following strategies to prevent dispensing errors based on the environment of the pharmacies: workload reduction, separate storage of similar drugs, additional labels for high-risk medications, support staff, and sufficient counseling time [33].

Regarding the interpretation of the study results, the following limitations should be considered. First, owing to the nature of data from voluntary reporting systems, under-reporting of MEs is an unknown but undoubtedly present limitation [34]. Additionally, there may be a tendency to report near misses, which are easy to detect and report, more frequently [22]. As a result, we could not estimate the actual prevalence of MEs in the community. Second, incompleteness of records, such as the omission of medication information, may affect the results of ME-related drugs. A total of 972 MEs (10.7%), including 145 harmful MEs, were excluded because the medications involved were not specified. Lastly, we could not objectively evaluate MEs based on the pharmacy environment and the characteristics of the prescribing physicians because those data were not available.

Despite these limitations, it is meaningful that this study is the first to systematically analyze MEs that occurred in the community in Korea and has presented a list of frequently reported medications using nationwide medication incidents reports. The findings of this study may help develop targeted interventions and can contribute to improving medication-related patient safety for patients residing in community settings by providing insight into the types of MEs and frequently involved medications.

## 5. Conclusions

The majority of community pharmacist-reported cases of MEs in Korea were accounted for by prescription errors, most of which were near misses, and the process during which harmful MEs most commonly occurred was dispensing. The most common types of prescribing MEs were “wrong drug” followed by “dosing error,” while “dosing error” was the most frequent dispensing and administration error, followed by “wrong drug”.

Identifying the environment in which MEs mainly occur and the major culprit medications that cause harm should be used as a fundamental basis to identify high-risk medications and establish guidelines for the safe management of medications specific to community care settings in Korea. Frequently reported medications in harmful incidents, including warfarin, levothyroxine, and glimepiride, should be made a priority for developing targeted strategies to prevent MEs.

## Figures and Tables

**Table 1:** Level of harm and type of medication errors by medication use process.

		Medication Use Process				
	Total	Prescribing	Dispensing	Administering	Others	Missing
	N (%)	N (%)	N (%)	N (%)	N (%)	N (%)
Total	9046	8098	605	171	72	100
	(100)	(89.5)	(6.7)	(1.9)	(0.8)	(1.1)
Level of harm						
Near miss	7985 (88.3)	7819 (86.4)	109 (1.2)	30 (0.3)	17 (0.2)	10 (0.1)
No harm	121 (1.3)	17 (0.2)	43 (0.5)	37 (0.4)	24 (0.3)	0
Mild harm	259 (2.9)	25 (0.3)	186 (2.1)	34 (0.4)	6 (0.1)	9 (0.1)
Moderate harm	135 (1.5)	16 (0.2)	101 (1.1)	10 (0.1)	0	8 (0.1)
Severe harm	6 (0.1)	0	6 (0.1)	0	0	0
Missing	540 (6.0)	222 (2.5)	160 (1.8)	60 (0.7)	25 (0.3)	73 (0.8)
Type of error						
Wrong drug	3815 (42.2)	3628 (40.1)	149 (1.6)	27 (0.3)	0	11 (0.1)
Dosing error	2437 (26.9)	2193 (24.2)	153 (1.7)	86 (1.0)	0	5 (0.1)
Wrong duration	1136 (12.9)	1136 (12.6)	0	0	0	0
Omission error	513 (5.7)	474 (5.2)	20 (0.2)	18 (0.2)	0	1 (0.0)
Wrong form/route	296 (3.3)	272 (3.0)	20 (0.2)	4	0	0
Wrong patient	157 (1.7)	116 (1.3)	41 (0.5)	0	0	0
Wrong count	58 (0.6)	0	58 (0.6)	0	0	0
Wrong storage	11 (0.1)	0	0	11 (0.1)	0	0
Expired medication	12 (0.1)	0	11 (0.1)	0	0	1 (0.0)
Mislabeling	14 (0.2)	0	14 (0.2)	0	0	0
Others	360 (4.0)	237 (2.6)	29 (0.3)	22 (0.2)	72 (0.8)	0
Missing	237 (2.6)	42 (0.5)	110 (1.2)	3 (0.0)	0	82 (0.9)

**Table 2:** Level of harm and type of errors of harmful medication errors by medication use process.

		Medication Use Process				
	Total	Prescribing	Dispensing	Administering	Others	Missing
	**N (%)**	**N (%)**	**N (%)**	**N (%)**	**N (%)**	**N (%)**
Harmful MEs	400	40	293	44	6	17
	(100)	(10.0)	(73.3)	(11.0)	(1.5)	(4.3)
Level of harm						
Mild harm	259 (64.8)	24 (6.0)	186 (46.5)	34 (8.5)	6 (1.5)	9 (2.3)
Moderate harm	135 (33.8)	16 (4.0)	101 (25.3)	10 (2.5)	0	8 (2.0)
Severe harm	6 (1.5)	0	6 (1.5)	0	0	0
Type of error						
Dosing error	124 (31.0)	9 (2.3)	83 (20.8)	27 (6.8)	0	5 (1.3)
Wrong drug	107 (26.8)	20 (5.0)	86 (21.5)	0	0	1 (0.3)
Wrong patient	22 (5.5)	3 (0.8)	19 (4.8)	0	0	0
Omission error	14 (3.5)	1 (0.3)	9 (2.3)	3 (0.8)	0	1 (0.3)
Wrong count	13 (3.3)	0	13 (3.3)	0	0	0
Mislabeling	6 (1.5)	0	6 (1.5)	0	0	0
Expired medication	6 (1.5)	0	5 (1.3)	0	0	1 (0.3)
Wrong storage	5 (1.3)	0	0	5 (1.3)	0	0
Wrong form/route	2 (0.5)	0	1 (0.3)	1 (0.3)	0	0
Others	24 (6.0)	0	13 (3.3)	5 (1.3)	6 (1.5)	0
Missing	77 (19.3)	7 (1.8)	58 (14.5)	3 (0.8)	0	9 (2.3)

**Table 3:** Frequently reported therapeutic classes and chemical substances associated with harmful medication errors.

Harmful Medication Errors (N = 255)						
Therapeutic Subgroup (ATC Level 2) ^a^		N	%	Chemical Substance	N	%
				**(ATC Level 5) ^a,b^**		
A10	Drugs used in diabetes	39	15.3	Warfarin	15	5.9
B01	Anti-thrombotic agents	21	8.2	Levothyroxine sodium	13	5.1
M01	Anti-inflammatory and anti-rheumatic products	19	7.5	Glimepiride	9	3.5
				Acetylsalicylic acid	5	2
H03	Thyroid therapy	17	6.7	Clonazepam	5	2
N05	Psycholeptics	16	6.3	Loxoprofen	5	2
C09	Agents acting on the renin-angiotensin system	14	5.5	Atorvastatin	4	1.6
N03	Anti-epileptics	11	4.3	Dexibuprofen	4	1.6
C10	Lipid-modifying agents	8	3.1	Metformin	4	1.6
N02	Analgesics	8	3.1	Naproxen	4	1.6
N06	Psychoanaleptics	8	3.1	Pregabalin	4	1.6

^a^ Sixteen harmful MEs related to anti-hypertensive agents in which the therapeutic subgroup (ATC level 2) could not be identified were excluded. ^b^ Fourteen harmful MEs related to anti-diabetic agents in which the chemical substance (ATC level 5) could not be identified were excluded.

## Data Availability

Restrictions apply to the availability of these data. Data were obtained from the Korean Pharmaceutical Association and are available at https://www.safepharm.or.kr/main.do (accessed on 25 October 2021) with the permission of the Korean Pharmaceutical Association.

## Supplementary Materials

The following supporting information can be downloaded at: https://www.mdpi.com/article/10.3390/medicina59010151/s1, Table S1: Types of medication errors and their inclusion; Table S2: Most reported therapeutic subgroups and chemical substances involving total MEs.
