# Supplementary material for: Analysis of Medication Errors Reported by Community Pharmacists in the Republic of Korea: A Cross-Sectional Study
Source: Medicina (Kaunas). 2023 Jan 12;59(1):151. doi: 10.3390/medicina59010151 (PMC9866739; doi:10.3390/medicina59010151)
Supplement: Supplementary file 1 [file medicina-59-00151-s001.zip › medicina-2083162-supplementary.pdf]

Table S1. Type of medication errors and their inclusion

Type	Inclusion
Wrong patient	Different patient
Wrong drug	Different drug, drug not indicated, known drug allergy or adverse drug reaction, contraindicated drug, therapeutic duplication, drug interaction
Dosing error	Improper dose (overdosage, under dosage, extra dose), wrong strength/concentration, wrong frequency, wrong time
Wrong duration	Prescribing of inappropriate duration
Wrong dosage form/route	Wrong dosage form, wrong route of administration
Wrong measuring/count	Dispensing of an incorrect quantity
Mislabeling	Wrong label/instruction
Wrong storage	Inappropriate storage
Omission/compliance error	Omitted medication or dose
Expired medication	Dispensing or administering a drug that has expired
Others	Quality-defecting drug

Table S2. Most reported therapeutic subgroups and chemical substances involving total MEs

Total MEs (N = 8074)						
Therapeutic subgroup (ATC level 2)		N	%	Chemical substance (ATC level 5)[a, b]	N	%
M01	ANTIINFLAMMATORY AND ANTIRHEUMATIC PRODUCTS	1518	18.8	loxoprofen	940	11.6
A02	DRUGS FOR ACID-RELATED DISORDERS	813	10.1	bepotastine	331	4.1
R06	ANTIHISTAMINES FOR SYSTEMIC USE	770	9.5	mosapride	193	2.4
R05	COUGH AND COLD PREPARATIONS	579	7.2	opium derivatives and expectorants	180	2.2
J01	ANTIBACTERIALS FOR SYSTEMIC USE	548	6.8	amoxicillin and beta-lactamase inhibitor	173	2.1
A03	DRUGS FOR FUNCTIONAL GASTROINTESTINAL DISORDERS	373	4.6	montelukast	154	1.9
R03	DRUGS FOR OBSTRUCTIVE AIRWAY DISEASES	318	3.9	dexibuprofen	144	1.8
N02	ANALGESICS	237	2.9	erdosteine	144	1.8
R01	NASAL PREPARATIONS	203	2.5	paracetamol	144	1.8
A10	DRUGS USED IN DIABETES	198	2.5	levocetirizine	140	1.7
